# The impact of deproteinized bovine bone particle size on histological outcomes in sinus floor elevation: a systematic review and meta-analysis

**DOI:** 10.1186/s40729-023-00502-1

**Published:** 2023-10-02

**Authors:** Xin Li, Shi-chen Lin, Shao-yu Duan

**Affiliations:** Department of Stomatology, Electric Power Teaching Hospital, Capital Medical University, No.1, Taipingqiao Xili, Fengtai District, Beijing, 100073 China

**Keywords:** Bio-Oss, Bone graft, Dental implants, Maxillary sinus, Sinus floor augmentation

## Abstract

**Objectives:**

The main purpose of this study was to evaluate whether large granular bovine bone can be as effective as small granular bovine bone in maxillary sinus floor elevation.

**Methods:**

A comprehensive online search of eligible articles was conducted using PubMed, EMBASE, Cochrane Library, Scopus, and Web of Science, and a systematic review and meta-analysis was performed from establishment to February, 2023. The outcome indicators were the percentage of connective tissue, the percentage of newly formed bone and the percentage of residual xenograft respectively. The meta-analysis was conducted by using the Stata 15.1 (Stata Conpernarn, USA) and Review Manager software5.4.1.

**Results:**

After careful screening and review, a total of 4 studies were included for systematic review and meta-analysis. The data were extracted to compare the histological performance of bovine bones with different particle sizes after maxillary sinus elevation. No significant differences were found in the percentage of connective tissue, the percentage of newly formed bone, and the percentage of residual xenograft.

**Conclusion:**

In this study, a systematically review of the previous literature showed that similar histological results were obtained for both large-particle bovine bone and small-particle bovine bone. Therefore, the large granular bovine bone and the small granular bovine bone were equally effective in maxillary sinus elevation. It is difficult to make conclusion from limited evidence from four studies. More clinical evidence was needed.

## Introduction

Due to maxillary sinus gasification and alveolar bone atrophy, many patients who requires maxillary posterior dental implants faces the problem of insufficient height of residual alveolar bone. In order to obtain sufficient healthy bone mass and good planting results, maxillary sinus floor elevation is a common clinical method to solve such problems [1–3]. For a long time, autologous bone has been considered as the gold standard for maxillary sinus augmentation. Even though, there are a number of disadvantages to the use of autologous bone for maxillary sinus enhancement. For example, there may be a need for hospitalization, the opening of a second surgical site, increased incidence of complications, and the inevitable tendency to absorb a lot [4, 5]. With the development of technology, bone incremental materials from various biological or synthetic origin are also increasingly being used to optimize surgical. Of all the major bone replacement grafts in used (allografts, xenografts, alloplats), the most clinical research are xenografts, such as bovine bone, pork bone, and horse bone. Among them, deproteinized bovine bone mineral has been widely used in sinus augmentation with comparable results and success rates [6, 7].

Bio-Oss® is one of the best documented bone graft materials manufactured in Switzerland for dental implant application [8, 9]. The product is divided into large particles and small particles according to particle size. The large particles are 1–2 mm, and the small particles are 0.25–1 mm. Bovine bone has demonstrated superior clinical presentation and favorable histological results over the years when used alone or in combination with autologous bone or other allografts [10–12]. At the same time, there is no evidence of disease transmission in terms of safety [13–15].

Previous studies have mainly focused on the physical and chemical properties of the graft materials, while relatively few research has been conducted on the effect of the particle size of the material itself on osteogenic. The relative merits of maxillary sinus elevation are unclear, although several studies have compared the clinical and histological outcomes of large and small granular bovine bone.

Based on the facts and background of previous studies, this article conducted a systematic review and meta-analysis to evaluate whether large granular bovine bone can be as effective as small granular bovine bone in maxillary sinus floor elevation. This study aims to provide some reference for future clinical application.

## Methods

This article investigated and reported results according to the Cochrane Manual and the Preferred Reporting Project for Systematic Reviews and meta-Analyses (PRISMA) statement. The protocol has been registered in PROSPERO(International Prospective Register of Systematic Reviews) a priori under registration number CRD42022379384.

### Search strategies

A comprehensive online search of eligible articles was conducted using PubMed, EMBASE, Cochrane Library, Scopus, and Web of Science. Search strings were created by using Boolean operators specifically combined with the keywords “AND” and “OR”. The search strings were as follows:([“maxillary sinus” OR “sinus”] AND [“floor elevation” OR “lift” OR “floor augmentation” OR “augmentation” OR “floor”]) AND (“xenograft” OR “bovine bone” OR “Bio-Oss” OR “inorganic bovine bone” OR “deproteinized bovine bone matrix”). There were no restrictions on the language of publication or the year of publication. The last search was conducted in February 2023.

### Eligibility criteria

#### Inclusion criteria

All randomized controlled clinical trials conducted in humans were taken into consideration. In addition, there were other aspects need to be considered: Firstly, maxillary sinus lift surgery was required in healthy patients with inadequate bone mass in the maxillary posterior region. Secondly, maxillary sinus lifting was performed using either large (1–2mm) or small (0.25 to 1 mm) Deproteinized Bovine Bone Mineral (DBBM) for bone increment.

#### Exclusion criteria

At the beginning of this study, all animal trials were excluded. More importantly, this study did not include studies that met several criteria: Firstly, meta-analysis, case reports, proceedings, retrospective and cohort studies, personal communications, and studies without control groups; Secondly, there were no corresponding evaluation index and duplicate studies.

### Data extraction

Two authors (XL and SCL) independently completed the literature screening. Eligible references were identified according to inclusion and exclusion criteria. Then data were extracted from the included literature, and the reasons for excluding other literature were recorded. The other authors checked the accuracy of the results.

### Quality assessment

Each included study was reviewed separately for risk of bias by XL and SCL authors, and the Cochrane Collaboration tool was used to assess risk of bias in randomized trials. The tool includes seven different domains. All domains and their included issues were evaluated and categorised as low risk, high risk, or representing unclear risk. After determining each domain, an overall estimation of the plausible risk of bias (low, moderate, or high) was performed for each selected study (low risk of bias: all domains were assessed as ‘low risk’; moderate risk of bias: one or more domains were assessed as ‘unclear’; high risk of bias: one or more domains were assessed as ‘high risk’). Differences of opinion between the two authors were resolved by discussion or negotiation with the other authors.The general chart of bias risk was made by Revman 5.4 software.

### Outcome measures and data analysis

The primary outcomes of this study were the percentage of connective tissue (CT), the percentage of newly formed bone (NFB), and the percentage of residual xenograft (RX). Authors XL and SCL used the Stata 15.1 (Stata Conpernarn, USA) and Review Manager software 5.4.1 for data synthesis. Weighted mean difference (WMD) and 95% confidence intervals (CI) were calculated to assess the overall efficacy of all included studies. Heterogeneity among studies was quantitatively assessed using the X^2^-based Q-test and I-squared (I^2^) statistic. The heterogeneity of the combined study was stronger when *P* < 0.1 and *I*^2^ > 50%, so the random effects model was used, otherwise the fixed-effects model was carried.

## Results

### Search results

**Fig. 1:**
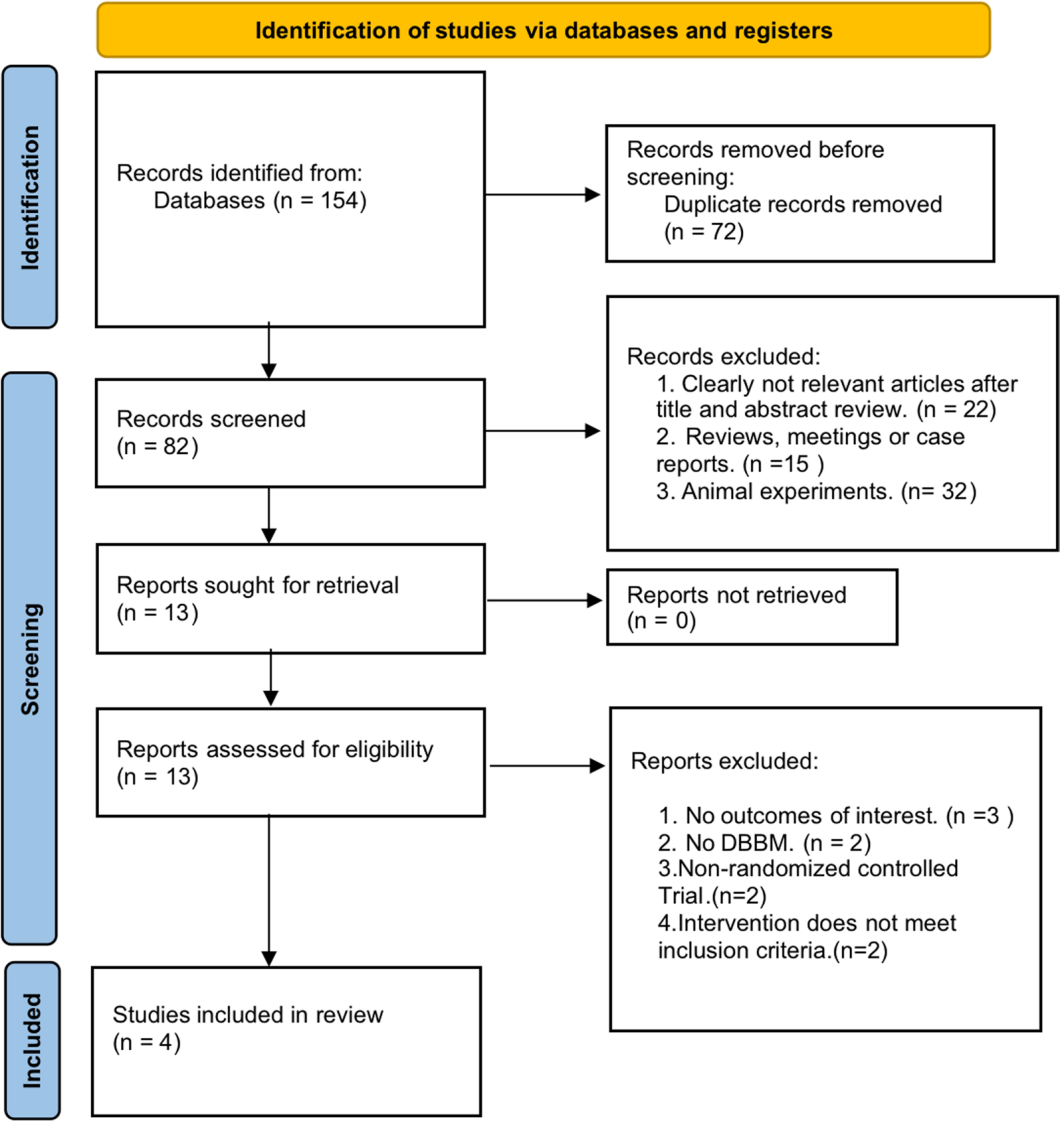
PRISMA Flow diagram of the screening selection process. PRISMA 2020 flow diagram for new systematic reviews which included searches of databases, registers and other sources

This study started with an electronic search through online databases, which produced 149 articles. After removing duplicates, 72 studies were retained by Endnote 20 software. After carefully reading of the full text of the remaining 72 articles and further assessment strictly according to the eligibility criteria, 68 publications were excluded because they did not meet these criteria. The flow chart and reasons for exclusion were shown in Fig. 1. Finally, a total of 4 studies were included in the systematic review.

### Study characteristics

The basic characteristics of the four studies included in the meta-analysis were summarized in in Table 1. All the four studies were Randomized Controlled Trials(RCTs). The number of patients in the studies ranged from 20 to 32 (the total number in all studies was 94). The number of implants in the large particle group ranged from 10 to 13 (total = 44) and in the small particle group ranged from 10 to 19 (total = 50).

**Table 1:** Characteristics of included studies

Author	Year	Nation	No of patient(T/LP/SP)			No of implants	Patient age(mean)	Sex M/F		RBH (mean ± SD)	Type of surgery	Implant placement	No of biopsies	Follow up period	Main outcomes
			T	LP	SP			M	F						
Chackartchi	2011	Israel	20	10	10	NR	54.25 (range:46–65)	6	4	L: 2.45 ± 1.46mm S: 1.95 ± 1.06mm	Lateral window	Delayed implantation	20	6–9 month	CT, NFB, RX
Tiziano	2013	Italy	22	11	11	NR	NR	NR		< 5mm	Lateral window	Delayed implantation	22	6–8 month	CT,NFB,RX
Molon	2018	Brazil	20	10	10	25	48.34 ± 12.83(range:30–65)	8	12	< 5mm	Lateral window	Delayed implantation	20	8 month	CT,NFB,RX
Paksinee	2022	Thailand	32	13	19	32	L: 56 ± 8.9 S: 58.57 ± 8.8	16	16	L: 3.18 ± 0.7mm S: 3.33 ± 0.87mm	Lateral window	Delayed implantation	32	6 month	CT,NFB,RX

*T* total, *LP* large particle DBBM, *SP* small particle DBBM, *RBH* residual bone height, *NR* not report, *CT* connective tissue(%), *NFB* newly formed bone(%), *RX* residual xenograft(%)

### Risk of bias

**Fig. 2:**
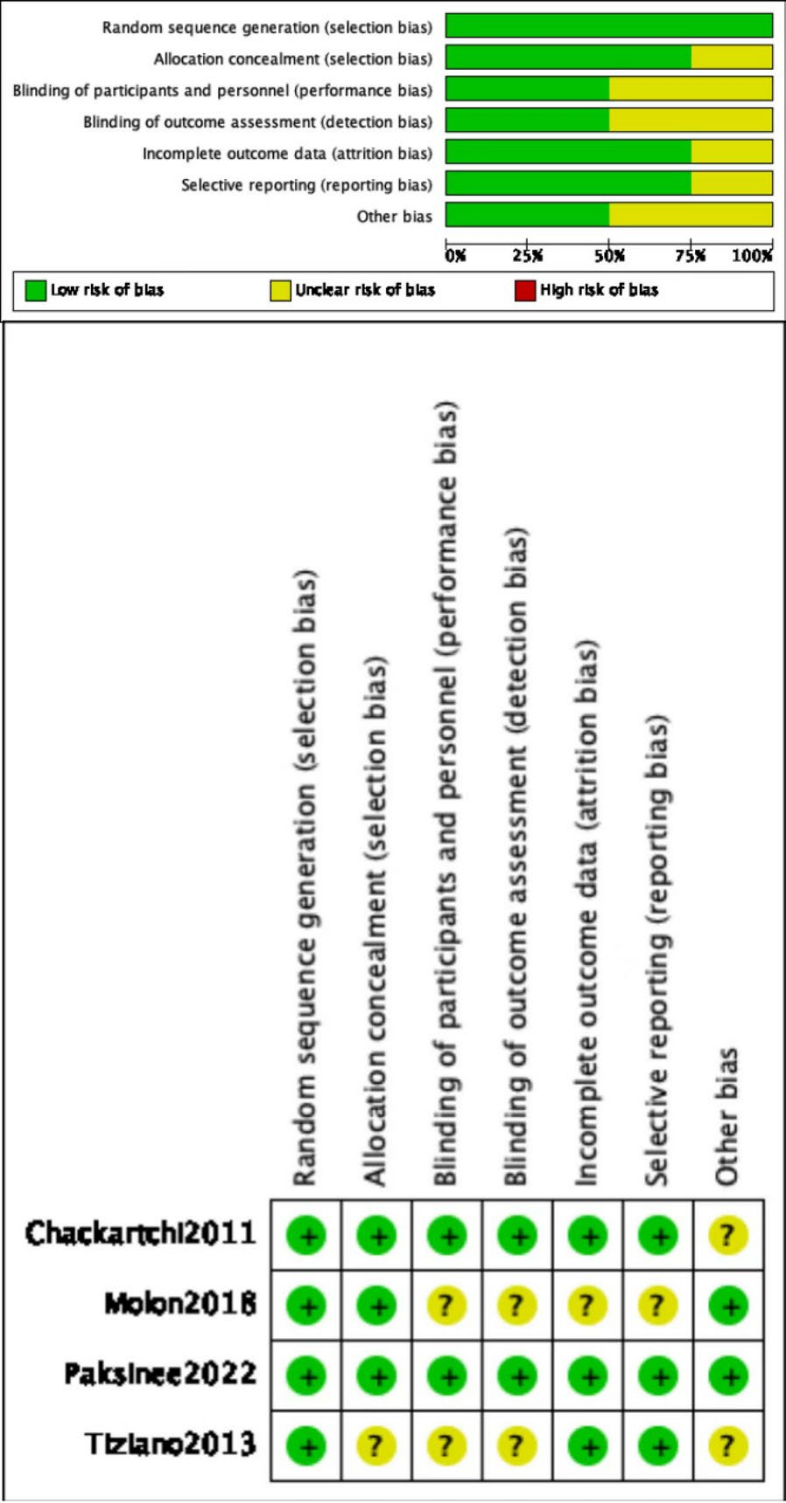
Risk of bias graph and summary

All four articles included in the study were declared to be randomised. All article mentioned allocation except for the study reported by Tiziano (2013). No paper perform blinding except for Chackartchi (2011) and Paksinee (2022). Except for Molon (2018), all included articles contained complete data, and no selective reporting was found. Chackartchi (2011) and Tiziano (2013) were considered to have unclear risk of other bias. Figure 2 presented the methodological quality assessment of the trials included in the review (Table 2).

**Table 2:** Outcome data of included studies

Author	Year	Percentage of CT		Percentage of NFB		Percentage of RX	
		LP	SP	LP	SP	LP	SP
Chackartchi	2011	39.14 (8.47)	37.42 (4.15)	27.14 (3.89)	28 (6.53)	33.71 (8.28)	34.57 (8.08)
Tiziano	2013	53.2 (11.5)	59.6 (9.9)	26.8 (9.6)	18.8 ( 4.7)	20 (9)	21.7 (10.5)
Molon	2018	23.8 (6.16)	30.4 (8.63)	36.7 (5.79)	36.1 (9.6)	38 (6.92)	32.4 (8.56)
Paksinee	2022	44.36 (26.7)	66.48 (20.97)	32.15 (14.04)	15.99 (14.12)	23.65 (17.18)	17.86 (16.42)

*LP* large particle DBBM, *SP* small particle DBBM, *CT* connective tissue(%), *NFB* newly formed bone(%), *RX* residual xenograft(%)

Data are mean(SD)

### Quantitative synthesis

#### Percentage of connective tissue

**Fig. 3:**
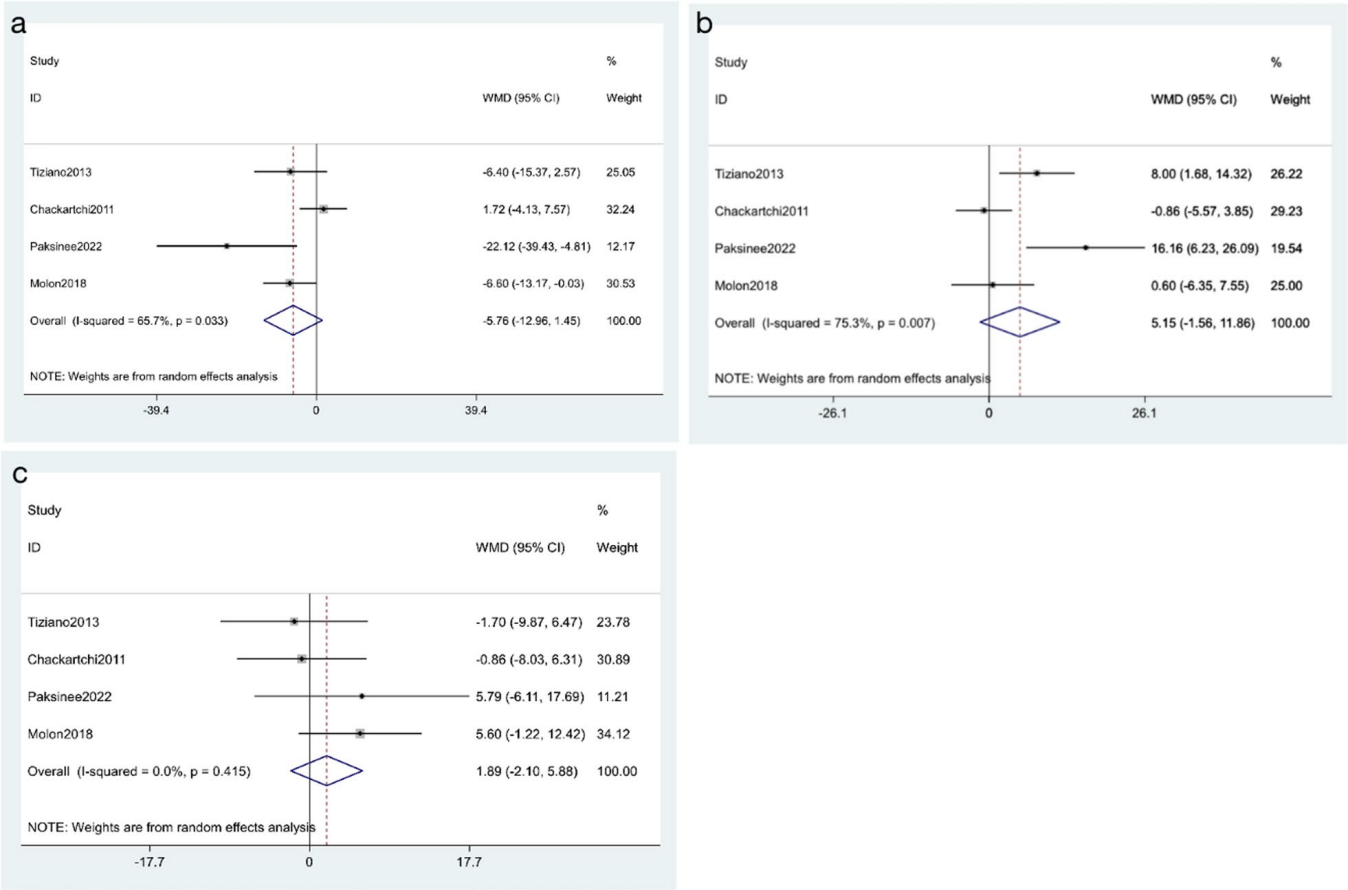
**a** Forest plots of connective tissue **b** Forest plots of newly formed bone **c** Forest plots of residual xenograft

All studies reported the percentage of connective tissue after surgery. A total of 94 patients were enrolled, including 44 cases in the small particle size of DBBM group and 50 cases in the large particle size of DBBM group. A random effect model was applied based on the presence of significant heterogeneity (*I*^2^ = 65.7%, *p* = 0.033). As shown in Fig. 3a, no significant increase in the percentage of connective tissue was observed among the included findings (WMD = − 5.76, 95% CI: − 12.96 to 1.45; *p* = 0.117).

#### Percentage of newly formed bone

All studies mentioned the percentage of newly formed bone. There were 94 patients in total, including 44 cases in the small particle size of DBBM group and 50 cases in the large particle size of DBBM group. A random effect model was applied based on a significant heterogeneity (*I*^2^ = 75.3%, *p* = 0.007). As shown in Fig. 3b, the results showed that the percentage of newly formed bone was non-significant between the large particle and the small particle (WMD = 5.15,95% CI: − 1.56 to 11.86; *p* = 0.132).

#### Percentage of residual xenograft

All studies provided the percentage of residual xenograft. There were 94 patients in total, including 44 cases in the small particle size of DBBM group and 50 cases in the large particle size of DBBM group. A fixed-effects model was applied, due to the *I*^2^ = 0.0% and *p* = 0.415. As shown in Fig. 3c, no difference in percentage of residual xenograft was observed between included studie (WMD = 1.89, 95% CI: − 2.10 to 5.88; *p* = 0.353).

## Discussion

This study compared the histological outcomes of DBBM with two different particle sizes during maxillary sinus floor elevation. It was concluded that there was no difference between the two DBBM preparations in histology and maxillary sinus floor lift by searching, screening and analyzing the previous literature. The two groups were similar in terms of percentage of connective tissue, the percentage of newly formed bone, and percentage of residual xenograft.

Multiple bone substitutes can be used in maxillary sinus elevation. According to the source of the materials, they can be divided into four categories: autografts, allografts, xenografts, and synthetic bone substitutes [16].

Autografts itself has osteogenic potential, and can be used as a scaffold for osteogenesis to play a role in osteoconduction and osteoinduction. The latter three types of bone substitutes lack osteoblastic cells, which mainly provide scaffolds for the formation of new bone and play the roles of osteoconduction and osteoinduction.

For maxillary sinus bone grafting with autogenous bone, a second surgical area needs to be opened to obtain sufficient autogenous bone. The opening of the second operative area not only increased the surgical trauma and operation time, but also the discomfort of the patients during and after the operation increased. Many patients have difficulty accepting autogenous bone grafting. Autogenous bone is rarely used in the treatment of maxillary sinus bone grafting [17, 18].

Allografts can be divided into three types: fresh/frozen bone, freeze-dried bone and demineralized freeze-dried bone. Among them, fresh/frozen bone had the greatest osteoinductive and osteoconductive potential. However, due to the risk of disease transmission, it is no longer in clinical application.

Synthetic bone substitutes refer to bioceramics or polymers made from natural materials or synthetic materials. Different synthetic bone substitutes have different physical and chemical properties and can be degraded in vivo or remain stable for a long time. However, as a scaffold material, it has no osteogenesis and osteoinducibility.

Xenogeneic bone refers to the bone graft substitutes derived from different species of biological individuals. The source is generally cattle, pigs, horses and other animals. The currently dominant product in the clinic is DBBM. DBBM is derived from natural calf bone and is a porous carbonate apatite crystal with bone conduction properties. Its physical and chemical properties are very similar to the structure of human bone tissue, and it retains the porous structure and trabecular bone of natural bone. It can provide a scaffold for the expansion of osteoblasts, and ensure the stability of blood clots and the regeneration of blood vessels. In the literature related to maxillary sinus elevation, DBBM as a bone augmentation material has involved the most clinical cases and the most complete data [19–22].

DBBM was a well-documented bone grafting material for maxillary sinus lift [23–26]. The most widely utilized commercial product in clinical practice was Bio-Oss with diameters ranging from 0.25 to 1 mm and 1–2 mm, respectively[27–29]. Bio-Oss was widely used because its characteristics, including its crystallinity and physicochemical properties, were very similar to those of human cancellous bone. DBBM acted as a scaffold and matrix to promote the migration of osteoblasts from the maxillary sinus wall to the graft material, and then increasing the ability of new bone formation [30–32]. There have been a number of studies using DBBM for maxillary sinus elevation and to evaluate the performance of bone healing from a histological perspective, but the application of DBBM with different particle sizes and maxillary sinus elevation has been limited and the results have been confusing. This is because maxillary sinus elevation with different sizes of DBBM results in completely different bone healing in only a few studies [33–36]. It was important to note that only four randomized controlled clinical trials have investigated the use of DBBM and maxillary fundus in different sizes.

A total of four literatures were included in this study. Chackartchi et al. [33] used large and small bovine bones separately in maxillary sinus floor lifting surgery. After a period of 6–9 months, they extracted bone samples from patients and found that both large and small bovine bones showed similar clinical and histological results. When comparing the application of large and small granular bovine bones in maxillary sinus floor elevation, Testori et al. [34] found that the large granular bovine bones produced more new bone than the small granular bovine bones in terms of histomorphometric results at 6–8 months after surgery. In addition, Molon et al. [35] conducted histomorphometric studies to stain the protein expression of osteocalcin, vascular endothelial growth factor and tartrate-resistant acid. The results showed no statistically significant difference between the large and the small, which suggesting that the size of DBBM did not affect the osteogenic effect during maxillary sinus elevation. Through a randomized controlled study, Kamolratanakul et al. [36] found that the application of large particles of DBBM in maxillary sinus floor elevation could induce more angiogenic expression and obtain more new bone. However, the clinical outcomes were similar in both groups. The reasons for their disagreement are multifaceted and the result of various factors, such as sample size, sample selection, population differences, differences in surgical techniques, and the way, location or time of collecting specimens.

In previous studies, bone specimens were obtained in different ways. Testori et al. [34] and Molon et al. [35] took bone specimens from the buccal side of the lateral wall of the maxillary sinus for analysis. On the contrary, Chackartchi et al. [33] and Kamolratanakul et al. [36] used a hollow bone drill to extract bone samples from the alveolar crest (the implant site) for analysis. The presence of partial autogenous cortical or cancellous bone in the bone specimens may affect the histological results, but this article believed that both methods of bone extraction were feasible, and the fact that autogenous bone may be included was unavoidable. Other limitations included the inability to control the size and morphology of the maxillary sinus itself and the size of its bulge. However, as the target of our evaluation was a complex multi-factor biological process, the experimental method and evaluation indicators were more important.

In modern medicine, immunohistochemical analysis can be independent of morphological observation, which can provide a broader overview of biological processes and directions of progress. In a previous report, ABB particles did not affect the expression of genes associated with bone remodeling and inflammation after a 6-month healing period. Histological evidence also suggested that DBBM particles were replaced by new bone formation and did not affect bone healing [37]. Similarly, Pereira et al. performed maxillary floor elevation 6 months after surgery using autogenous bone and DBBM, and they found similar histopathological and immunohistochemical evaluations of RUNX2 and vascular endothelial growth factor (VEGF) [38]. These data suggested that bone remodeling and neovascularization occur at least 6 months after surgery when DBBM was used for external maxillary sinus elevation, which explained why the experimental period of maxillary sinus lift was at least 6 months. Moreover, the use of DBBM did not inhibit the expression of genes related to bone remodeling induction. It is worth noting that the four studies included in this paper also had an observation period of more than 6 months, which revealed the reliability of DBBM in clinical application.

Although both large and small particles used for grafting maxillary sinus lifting after surgery have similar histological results, they have significant advantages and disadvantages from the perspective of clinical application. For large particles, it can safely reduce the amount of biomaterial filling the maxillary sinus without affecting the graft volume, so more space could be obtained for implantation. Another important aspect was that the surgical time can also be shortened due to the reduction in the size of the graft. Conversely, the use of large particles also increased the amount of void space in the whole area, thus in turn increased the risk of infection. For small particles, its application allowed for a better grasp of the space and volume of bone graft, based on the size of the maxillary sinus, the number of implants needed, the anatomy of the maxillary sinus and other factors. On the other hand, the application of DBBM with small particles for suitable patients can not only reduce the use of materials, but also reduce the consumption of patients while achieving the same results as the large particles.

When it comes to implant success rates, even in cases of perfect bone condition, objective factors such as general health must be considered, not to mention the complexities involved in maxillary sinus elevation. The implant stability is regarded as a crucial factor for successful osseointegration and serves as one of the most commonly employed indicators to predict implant stability. There are numerous factors that impact the stability of implants, including but not limited to overall physical health, bone density and quantity, implant surface design, among others. There is little evidence suggests that the utilization of various bone substitutes has impact on implant stability [39–42].

Even if the stability of the implant is not significantly affected by the bone graft material, it does not imply that the bone substitutes are insignificant. No matter which type of bone graft material is used to support the maxillary sinus mucosa, it can effectively maintain the stability of the osteogenic space, particularly when utilizing a small diameter implant tip. Additionally, the bone graft material can disperse pressure on the maxillary sinus mucosa, preventing secondary infections caused by maxillary sinus perforation or collapse of the maxillary sinus mucosa that could reduce osteogenic space.

In conclusion, this study systematically reviewed the previous literature and found that both large-particle DBBM and small-particle DBBM could achieve similar histological results in the following three aspects during maxillary sinus elevation: connective tissue, newly formed bone, and residual xenograft. It can draw a conclusion from the above that the large granular bovine bone and the small granular bovine bone were equally effective in maxillary sinus elevation, which provided a valuable reference for clinical application.It is difficult to make conclusion from limited evidence from four studies. More clinical evidence was needed.

## Data Availability

The datasets used and/or analysed during the current study available from the corresponding author on reasonable request.

## References

[1] Danesh-Sani SA, Loomer PM, Wallace SS (2016). A comprehensive clinical review of maxillary sinus floor elevation: anatomy, techniques, biomaterials and complications. Br J Oral Maxillofac Surg.

[2] Bathla SC, Fry RR, Majumdar K (2018). Maxillary sinus augmentation. J Indian Soc Periodontol.

[3] Farina R, Franzini C, Trombelli L, Simonelli A (2000). Minimal invasiveness in the transcrestal elevation of the maxillary sinus floor: a systematic review. Periodontol.

[4] Mardinger O, Chaushu G, Sigalov S, Herzberg R, Shlomi B, Schwartz-Arad D (2011). Factors affecting changes in sinus graft height between and above the placed implants. Oral Surg Oral Med Oral Pathol Oral Radiol Endod.

[5] De Santis E, Lang NP, Ferreira S, Rangel Garcia I, Caneva M, Botticelli D (2017). Healing at implants installed concurrently to maxillary sinus floor elevation with Bio-Oss® or autologous bone grafts. A histo-morphometric study in rabbits. Clin Oral Implants Res.

[6] Trimmel B, Gede N, Hegyi P, Szakács Z, Mezey GA, Varga E, Kivovics M, Hanák L, Rumbus Z, Szabó G (2021). Relative performance of various biomaterials used for maxillary sinus augmentation: a Bayesian network meta-analysis. Clin Oral Implants Res.

[7] Starch-Jensen T, Mordenfeld A, Becktor JP, Jensen SS (2018). Maxillary sinus floor augmentation with synthetic bone substitutes compared with other grafting materials: a systematic review and meta-analysis. Implant Dent.

[8] Lai VJ, Michalek JE, Liu Q, Mealey BL (2020). Ridge preservation following tooth extraction using bovine xenograft compared with porcine xenograft: a randomized controlled clinical trial. J Periodontol.

[9] Starch-Jensen T, Aludden H, Hallman M, Dahlin C, Christensen AE, Mordenfeld A (2018). A systematic review and meta-analysis of long-term studies (five or more years) assessing maxillary sinus floor augmentation. Int J Oral Maxillofac Surg.

[10] Galindo-Moreno P, Abril-García D, Carrillo-Galvez AB, Zurita F, Martín-Morales N, O'Valle F, Padial-Molina M (2022). Maxillary sinus floor augmentation comparing bovine versus porcine bone xenografts mixed with autogenous bone graft. A split-mouth randomized controlled trial. Clin Oral Implants Res.

[11] Pereira RDS, Bonardi JP, Ouverney FRF, Campos AB, Griza GL, Okamoto R, Hochuli-Vieira E (2020). The new bone formation in human maxillary sinuses using two bone substitutes with different resorption types associated or not with autogenous bone graft: a comparative histomorphometric, immunohistochemical and randomized clinical study. J Appl Oral Sci.

[12] Alayan J, Ivanovski S (2018). A prospective controlled trial comparing xenograft/autogenous bone and collagen-stabilized xenograft for maxillary sinus augmentation-Complications, patient-reported outcomes and volumetric analysis. Clin Oral Implants Res.

[13] Sogal A, Tofe AJ (1999). Risk assessment of bovine spongiform encephalopathy through bone graft material derived from bovine bone used for dental applications. J Periodontol.

[14] Wenz B, Oesch B, Horst M (2001). Analysis of the risk of transmitting bovine spongiform encephalopathy through bone grafts derived from bovine bone. Biomaterials.

[15] Kim Y, Nowzari H, Rich SK (2013). Risk of prion disease transmission through bovine-derived bone substitutes: a systematic review. Clin Implant Dent Relat Res.

[16] Velich N, Nemeth Z, Toth C (2004). Long-term results with different bone substitutes used for sinus floor elevation. J Craniofac Surg.

[17] Browaeys H, Bouvry P, Bruyn De H (2007). A literature review on biomaterials in sinus augmentation procedures. Clin Implant Dent Relat Res.

[18] Bornstein MM (2008). Performance of dental implants after staged sinus floor elevation procedures: 5-year results of a prospective study in partially edentulous patients. Clin Oral Implant Res.

[19] Cho YD, Namgung DJ, Kim KH, Kim S, Seol YJ, Lee YM, Ku Y (2022). Long-term human histologic evaluation of sinus bone augmentation and simultaneous implant placement. Int J Periodontics Restorative Dent.

[20] Dragonas P, Prasad HS, Yu Q, Mayer ET, Fidel PL (2023). Bone regeneration in maxillary sinus augmentation using advanced platelet-rich fibrin (A-PRF) and plasma rich in growth factors (PRGF): a pilot randomized controlled trial. Int J Periodontics Restorative Dent.

[21] Merli M, Moscatelli M, Merli M, Mariotti G, Pagliaro U, Nieri M (2022). Lateral sinus floor elevation in the severely atrophied maxilla: concentrated growth factors versus bone substitutes. A controlled clinical trial. Int J Periodontics Restorative Dent.

[22] Merli M, Moscatelli M, Mariotti G, Pagliaro U, Merli M, Nieri M (2021). Use of autogenous bone versus deproteinised bovine bone matrix in one-stage lateral sinus floor elevation in severely atrophied maxillae: a 7-year randomised controlled trial. Int J Oral Implantol (Berl).

[23] Younes F, Cosyn J, De Bruyckere T, Cleymaet R, Eghbali A (2019). A 2-year prospective case series on volumetric changes, PROMs, and clinical outcomes following sinus floor elevation using deproteinized bovine bone mineral as filling material. Clin Implant Dent Relat Res.

[24] Irdem HO, Dolanmaz D, Esen A, Ünlükal N, Şimsek S (2021). Evaluation of the effectiveness of liquid platelet-rich fibrin and deproteinized bovine bone mineral mixture on newly formed bone in maxillary sinus augmentation: a split-mouth, Histomorphometric Study. Niger J Clin Pract.

[25] Taschieri S, Moses O, Dellavia C, Canciani E, Nemcovsky C, Francetti L, Corbella S (2021). Comparative study of deproteinized bovine bone mineral and bovine bone mineral enriched with a polymer and gelatin in maxillary sinus floor elevation procedures. Int J Periodontics Restorative Dent.

[26] Liu Y, Wang J, Chen F, Feng Y, Xie C, Li D (2020). A reduced healing protocol for sinus floor elevation in a staged approach with deproteinized bovine bone mineral alone: a randomized controlled clinical trial of a 5-month healing in comparison to the 8-month healing. Clin Implant Dent Relat Res.

[27] Wu Y, Xiao P, Xu A, He F (2022). Radiographic assessment of deproteinized bovine bone mineral (DBBM) and collagen-stabilized DBBM for transalveolar sinus floor elevation:a 2-year retrospective cohort study. Clin Implant Dent Relat Res.

[28] Da Silva HF, Goulart DR, Sverzut AT, Olate S, de Moraes M (2020). Comparison of two anorganic bovine bone in maxillary sinus lift: a split-mouth study with clinical, radiographical, and histomorphometrical analysis. Int J Implant Dent.

[29] Martiniano CRQ, Valadas LAR, Lins do Carmo Filho JR, Alves APNN, Leitão Lotif MA, Sotto-Maior BS, Dantas TCFB, Rodrigues LLFR, Francischone CE. A comparative histomorphometric analysis of two biomaterials for maxillary sinus augmentation: a randomized clinical, crossover, and split-mouth study. Evid Based Complement Alternat Med. 2022;2022:4577148. 10.1155/2022/4577148. 10.1155/2022/4577148 PMC9184224 35692573

[30] Xu AT, Qi WT, Lin MN, Zhu YH, He FM (2020). The optimization of sintering treatment on bovine-derived bone grafts for bone regeneration: in vitro and in vivo evaluation. J Biomed Mater Res B Appl Biomater.

[31] Dumitrescu CR, Neacsu IA, Surdu VA, Nicoara AI, Iordache F, Trusca R, Ciocan LT, Ficai A, Andronescu E (2021). Nano-hydroxyapatite vs. xenografts: synthesis, characterization, and in vitro behavior. Nanomaterials (Basel)..

[32] Gashtasbi F, Hasannia S, Hasannia S, Mahdi Dehghan M, Sarkarat F, Shali A (2020). Comparative study of impact of animal source on physical, structural, and biological properties of bone xenograft. Xenotransplantation.

[33] Chackartchi T, Iezzi G, Goldstein M (2011). Sinus floor augmentation using large (1–2 mm) or small (0.25–1 mm) bovine bone mineral particles: a prospective, intra-individual controlled clinical, micro-computerized tomography and histomorphometric study. Clin Oral Implants Res.

[34] Testori T, Wallace SS, Trisi P, Capelli M, Zuffetti F, Del Fabbro M (2013). Effect of xenograft (ABBM) particle size on vital bone formation following maxillary sinus augmentation: a multicenter, randomized, controlled, clinical histomorphometric trial. Int J Periodontics Restorative Dent.

[35] de Molon RS, Magalhaes-Tunes FS, Semedo CV (2019). A randomized clinical trial evaluating maxillary sinus augmentation with different particle sizes of demineralized bovine bone mineral: histological and immunohistochemical analysis. Int J Oral Maxillofac Surg.

[36] Kamolratanakul P, Mattheos N, Yodsanga S, Jansisyanont P (2022). The impact of deproteinized bovine bone particle size on histological and clinical bone healing outcomes in the augmented sinus: a randomized controlled clinical trial. Clin Implant Dent Relat Res.

[37] Suwanwela J, Puangchaipruk D, Wattanasirmkit K, Kamolratanakul P, Jansisyanont P (2017). Maxillary sinus floor augmentation using xenograft: gene expression and histologic analysis. Int J Oral Maxillofac Implants.

[38] Pereira RDS, Menezes JD, Bonardi JP, Griza GL, Okamoto R, Hochuli-Vieira E (2017). Histomorphometric and immunohistochemical assessment of RUNX2 and VEGF of Biogran™ and autogenous bone graft in human maxillary sinus bone augmentation: a prospective and randomized study. Clin Implant Dent Relat Res.

[39] Sezavar M, Bohluli B, Chehelamiran M, Danesh S, Shahriar A, Malekpour Z (2018). Comparison of implant stability in sinus lift surgery using autogenous versus allogeneic bone grafts. J Res Dentomaxillofac Sci.

[40] Khaled H, Atef M, Hakam M (2019). Maxillary sinus floor elevation using hydroxyapatite nano particles vs tenting technique with simultaneous implant placement: a randomized clinical trial. Clin Implant Dent Relat Res.

[41] Kudoh K, Fukida N, Kasugai S (2019). Maxillary sinus floor augmentation using low-crystalline carbonate apatite granules with simultaneous implant installation: first-in-human clinical trial. J Oral Maxillofac Surg..

[42] Dursun CK, Dursun E, Eratalay K, Orhan K, Tatar I, Baris E, Tözüm TF (2016). Effect of porous titanium granules on bone regeneration and primary stability in maxillary sinus: a human clinical, histomorphometric, and microcomputed tomography analyses. J Craniofac Surg.

